# Evaluating the stability of disulfide bridges in proteins: a torsional potential energy surface for diethyl disulfide

**DOI:** 10.1080/08927020701361876

**Published:** 2007-08-15

**Authors:** N. L. Haworth, J. E. Gready, R. A. George, M. A. Wouters

**Affiliations:** † Structural and Computational Biology Program, Victor Chang Cardiac Research Institute, Sydney, NSW 2010, Australia; ‡ Computer-Chemie-Centrum, University of Erlangen-Nuremberg, Nägelsbachstrasse 25, 91052 Erlangen, Germany; ¶ Computational Proteomics Group, John Curtin School of Medical Research, GPO Box 334, Canberra City ACT 2601, Australia; § School of Biotechnology and Biomolecular Sciences, School of Medical Sciences, University of New South Wales, Sydney, NSW, Australia

**Keywords:** Diethyl disulfide, Potential energy surface, Disulfide bond, Stability prediction, Redox activity, Arsenate reductase

## Abstract

Disulfide bonds formed by the oxidation of cysteine residues in proteins are the major form of intra- and inter-molecular covalent linkages in the polypeptide chain. To better understand the conformational energetics of this linkage, we have used the MP2(full)/6-31G(d) method to generate a full potential energy surface (PES) for the torsion of the model compound diethyl disulfide (DEDS) around its three critical dihedral angles (χ_2_, χ_3_, χ_2_′). The use of ten degree increments for each of the parameters resulted in a continuous, fine-grained surface. This allowed us to accurately predict the relative stabilities of disulfide bonds in high resolution structures from the Protein Data Bank. The MP2(full) surface showed significant qualitative differences from the PES calculated using the Amber force field. In particular, a different ordering was seen for the relative energies of the local minima. Thus, Amber energies are not reliable for comparison of the relative stabilities of disulfide bonds. Surprisingly, the surface did not show a minimum associated with χ_2_ ∼ − 60°, χ_3_ ∼ 90, χ_2_′ ∼ − 60°. This is due to steric interference between Hα atoms. Despite this, significant populations of disulfides were found to adopt this conformation. In most cases this conformation is associated with an unusual secondary structure motif, the cross-strand disulfide. The relative instability of cross-strand disulfides is of great interest, as they have the potential to act as functional switches in redox processes.

## 1. Introduction

Disulfide bonds between oxidised cysteine residues are generally viewed as structurally stabilising elements in proteins. However,a new role for a subset of disulfides as redox switches is emerging. Redox switching of disulfide bonds has been demonstrated in both reversible and irreversible redox regulation of proteins. Reversible systems include those involved in redox signalling such as the peroxide sensor, OxyR, where disulfide-bond formation activates the transcription factor in response to oxidative stress [1]. Irreversible redox regulation mediated by disulfide reduction and subsequent irreversible conformational change has also been described. For example, reduction of disulfides and subsequent cleavage of protein chains has been demonstrated in ovotransferrin and plasmin [2,3] and is likely to be an important regulatory mechanism for many other proteins.

**Figure 1:**
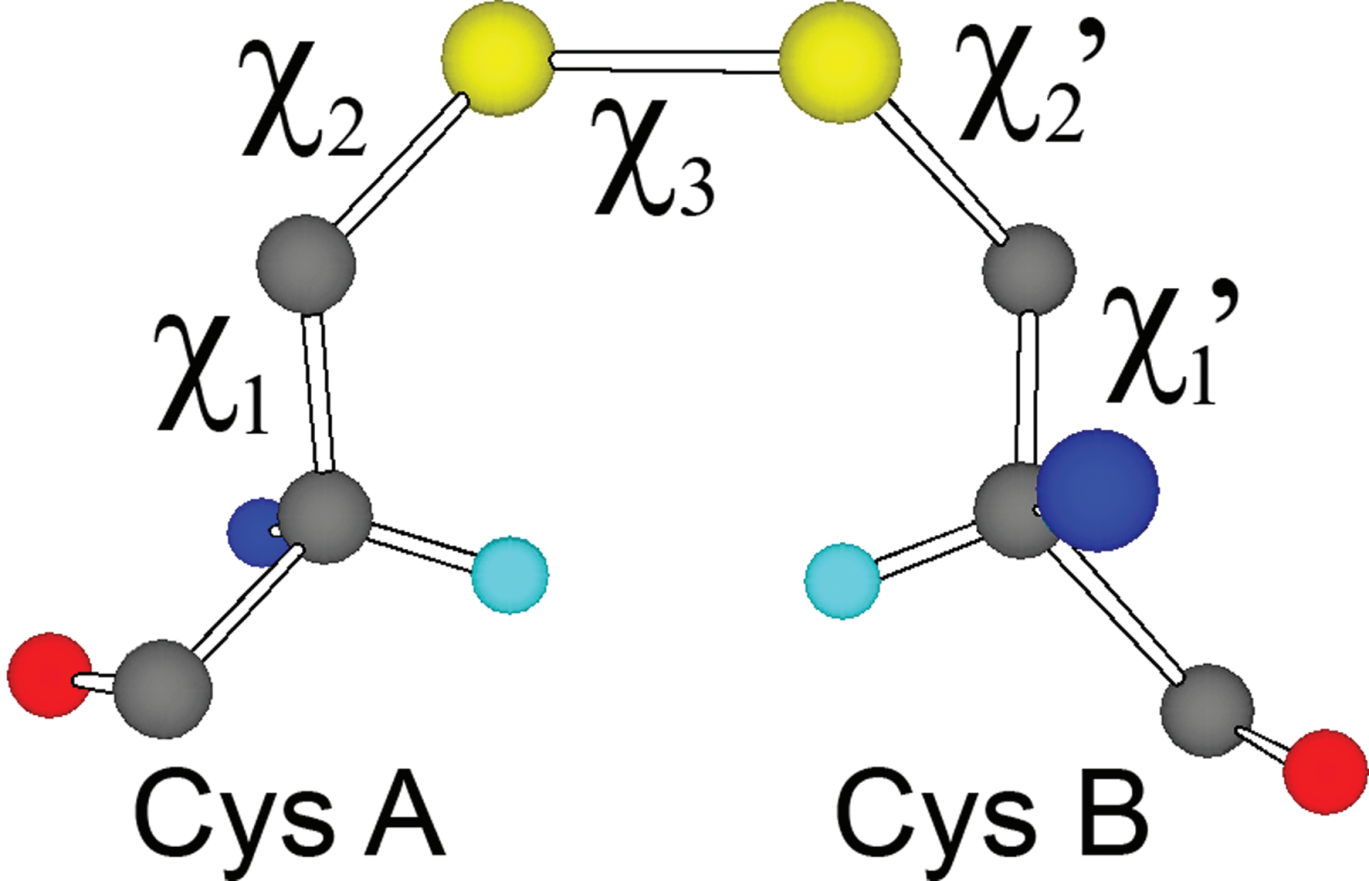
An example of a disulfide-bond conformation (G′GG′) between two cysteine residues showing the five critical torsion (dihedral) angles. Hα atoms are shown in cyan.

In principle it should be possible to differentiate between redox-active and structurally-stabilising disulfides by analysis of protein structures and ultimately protein sequences. Our previous studies have investigated high disulfide torsional energies as indicators of redox activity as well as identifying structural motifs associated with redox activity [4,5]. Torsional strain on the disulfide bond is imposed by the geometric constraints of the protein-fold. As the force constants for torsion around the dihedral angles are much lower than for the stretching and compressing of bond lengths and bond angles, this strain is expected to be mostly accommodated via torsion of the five critical dihedral angles of the disulfide (figure 1). From analysis of these torsional angles in high resolution X-ray structures of proteins, it should be possible to make a good prediction of the strain of a disulfide bond, and thus determine how likely it is to undergo redox processes.

In previous work [4], we estimated the stability of a disulfide bond using the torsional potential energy function [6] from the Amber force field [7]:

E(kcal mol−1)=2(1+cos 3χ1)+2(1+cos 3χ1′)+(1+cos 3χ2)+(1+cos 3χ2′)+3.5(1+cos 2χ3)+0.6(1+cos 3χ3)

While this function has the right general form, it clearly does not take into account the steric interactions within the system. This results in a potential energy surface (PES) in which all of the local minima are, incorrectly, predicted to be equally stable. The function is, therefore, not accurate when comparing disulfide stabilities in real systems. A better description of the relative stabilities which includes steric effects can be found using full Amber calculations, i.e. including non-bonded terms, but these have also been reported to give inaccurate results [8], a claim which will be investigated in this work.

In 1994, Görbitz [9] attempted to improve the understanding of the conformational preferences of disulfide bridges by performing *ab initio* calculations on the model compound, diethyl disulfide (DEDS). This model system is too small to allow investigation of the χ_1_ and χ_1_′ dihedral angles, however, it is possible to determine how the energy of the system changes as χ_2_, χ_2_′ and χ_3_ are varied. This is where the majority of the inaccuracy in the Amber function is expected to lie. Görbitz employed both the Hartree–Fock (HF) and MP2(full) levels of theory, with basis sets up to 6-311G(2d,p), to investigate the relative stabilities of the minima and saddle points on the PES defined by χ_2_, χ_2_′ and χ_3_. The dihedral angles χ_2_ and χ_2_′ were found to prefer values of +60, −60 and 180° (which Görbitz labelled G, G′ and T, respectively), while χ_3_ preferred to be +90 or −90 (G and G′). This gave rise to six symmetrically distinct minima and eight distinct saddle points. Görbitz found that methods which involved electron correlation (MP2(full)) were necessary to correctly predict the relative stabilities of the critical points. At the MP2(full) level, the preferred conformation was found to be GGG (χ_2_ ∼ 60°, χ_3_ ∼ 90°, χ_2_′ ∼ 60°), followed by G′GG, TGG, G′GT, TGT and G′GG′. With HF, GGG was correctly predicted to be the most stable, however, TGG and TGT were not appreciably higher in energy.

While Görbitz's calculations were state-of-the-art at the time they were reported, there have been significant developments both in quantum chemical methods and in computational power. In particular, density functional methods (such as B3LYP [10–14]) have become widely accepted; and schemes, such as G3 [15] and G3X [16], have been developed for performing highly accurate calculations at relatively low computational cost.

In order to distinguish between disulfide bridges that are simply performing a structural role and those which are likely to be redox active, we need to be able to accurately predict the relative stabilities of disulfide bonds. It is necessary not only to understand the relative stability of the torsional minima but also to have a good description of the entire PES. Like Görbitz, we have chosen to focus on the three central dihedral angles, χ_2_, χ_2_′ and χ_3_, thus reducing a very large five-dimensional problem to a far more tractable three dimensions. We expect that the torsion around the carbon–carbon bonds, χ_1_ and χ_1_′, should be relatively well described in the Amber force field. Also, χ_1_ and χ_1_′ do not, in general, show significant deviation from their optimal values. The goal of this work, therefore, is to create a new three-dimensional potential energy surface (3D-PES) for the torsion of DEDS around the χ_2_, χ_2_′ and χ_3_ dihedral angles.

## 2. Methods

Benchmarking calculations were initially carried out in order to determine the most reliable and cost effective level of theory with which to determine the 3D-PES. Reference energies for the minima and low lying saddle points were calculated using the G3X method [16]. G3X involves optimising the geometry at the B3LYP/6-31G(2df,p) level of theory, then performing a single-point energy calculation with QCISD(T)/6-31G(d). Further calculations are then used to correct this energy for the effects of including diffuse and higher polarisation functions in the basis set (at the MP4 level), including correlation of core electrons and even higher polarisation functions (at the MP2 level) and including g functions on the sulfur atom (at the HF level). A G3X electronic energy is thus obtained. Usually the zero-point energy is also included (calculated using B3LYP/6-31G(2df,p)), however, in this case it was omitted as we needed to use the reference energies to find the level of theory which gave the best possible prediction of the *electronic* energy of DEDS. The additional calculation of zero-point energies at every point of our 3D-PES would be far too computationally expensive. G3X is reported to yield an accuracy of ±4 kJ mol^−1^ for the calculation of heat of formation from atomisation energies. It is, therefore, expected to be highly accurate for the prediction of the relative energies of the DEDS minima and saddle points.

The G3X electronic energies were then compared with the energies from fully optimised calculations for each of the critical points, calculated using HF/6-31G(d), B3LYP/6-31G(d), B3LYP/6-31G(2df,p) and MP2(full)/6-31G(d). Amber energies for each of these critical points were also calculated for comparison.

The 3D-PES was calculated at the MP2(full)/6-31G(d) level of theory. Energies were calculated at ten degree increments in χ_2_, χ_2_′ and χ_3_ to give the full 3D grid. As Amber calculations for DEDS were relatively cheap to perform, a similar 3D-PES was created using Amber for comparison. This was only done for χ_3_ values between 60 and 130° as these represent the χ_3_ values adopted by over 99% of the high resolution X-ray structures found in the Protein Data Bank (vide infra).

The small increments used to calculate the PES resulted in a surface which was sufficiently fine grained that a simple linear interpolation could be used to predict the energies of disulfides with a given set of χ_2_, χ_2_′ and χ_3_ dihedral angles. This methodology was used to predict the relative stabilities of the disulfides in our database of high resolution disulfides in the Protein Data Bank [17]. Please see Ref. [18] for details of how this database was constructed.

All calculations were performed using the Gaussian 03 suite of programmes [19]. This suite includes the current version of Amber, version 9. Calculations were performed on the SC and LC computer clusters of the Australian Partnership of Advanced Computing (APAC) National Facility and on the local computer cluster of the Computer Chemie Centrum, Erlangen, Germany.

## 3. Results and discussion

### 3.1 Benchmark calculations

The results of the benchmarking calculations are shown in table 1. Energies are reported relative to the energy of the GGG conformation (the minimum on the PES at the G3X level of theory). Mean and RMS deviations from the G3X results are reported for each of the levels of theory investigated, together with the maximum deviation seen. The HF and MP2(full) results are the same as those described by Görbitz [9] and have been included here for comparison. Very little deviation was seen between the geometries of the different conformations at the different levels of theory.

**Table 1:** Relative energies (kJ mol2 1 ) of the diethyl disul.de minima and low energy saddle points at various levels of theory. Also included are mean, RMS and maximum deviations from the highest level of theory, G3X.

Conformation (χ_a_, χ_3_, χ_b_)*,†	Amber	HF 6-31G(d)	B3LYP 6-31G(d)	B3LYP 6-31G(2df,p)	MP2(full) 6-31G(d)	G3X
GGG (60°, 90°, 60°)	0.0	0.0	0.0	0.0	0.0	0.0
GGG′ (60°, 90°, −60°)	1.0	1.9	1.5	1.5	1.4	0.8
GGT (60°, 90°, 180°)	−0.3	0.1	1.6	1.7	2.1	2.3
G′ GT (−60°, 90°, 180°)	0.8	2.0	2.8	3.0	3.4	3.1
TGT (180°, 90°, 180°)	−0.6	0.3	2.9	3.1	4.2	4.8
G′ GG′ (−60°, 90°, −60°)	6.3	7.6	6.2	6.0	7.3	6.7
GGS (60°, 90°, 120°)	8.4	6.9	6.3	6.0	8.2	7.5
G′ GS (−60°, 90°, 120°)	8.3	7.4	6.7	6.4	7.9	6.7
GGS′ (60°, 90°, −120°)	7.5	7.7	6.9	6.7	8.6	8.3
TGS (180°, 90°, 120°)	8.1	6.9	7.6	7.5	10.2	9.9
TGS′ (180°, 90°, −120°)	7.4	7.7	8.3	8.2	10.6	10.6
G′ GS′ (−60°, 90°, −120°)	11.7	10.9	8.4	8.1	11.1	10.0
Mean deviation from G3X	−1.0	−0.9	−1.0	−1.0	0.4	
RMS deviation from G3X	2.3	2.0	1.3	1.4	0.6	
Max deviation from G3X	−5.4	−4.5	−2.3	−2.4	1.2	

* Dihedral angles shown are the average/minimum energy values for each conformation. See figure 1 for dihedral angle definitions.

† Due to the symmetry of the system, χ_a_ and χ_b_ can represent either χ_2_ or χ_2_′. That is, the conformation with χ_2_ = 60°, χ_3_ = 90°, χ_2_′ = −60° is identical in energy to the conformation with χ_2_ = −60°, χ_3_ = 90°, χ_2_′ = 60°.

The Amber energies in column 2 show the most significant deviation from the benchmarks, both in terms of the RMS deviation (2.3 kJ mol^−1^) and in having the largest discrepancy for any one configuration (TGT being predicted to be 5.4 kJ mol^−1^ too stable relative to GGG). Most importantly, confirming earlier reports [8], the order of the stabilities of the critical points is not consistent with the benchmark G3X results. The comparison between the HF results and the new G3X benchmarks was similarly poor, with an RMS of 2.0 kJ mol^−1^ and a maximum deviation of 4.5 kJ mol^−1^. The order of the critical points was better than observed for the Amber force field calculations but was still not correct in some cases.

The density functional results, with both the 6-31G(d) and 6-31G(2df,p) basis sets, also showed surprisingly poor agreement with the benchmark relative energies. Although the order of the minima was now correctly described, both methods predicted the GGG′ and GGT conformations to be roughly equal in energy, and likewise the G′GT and TGT minima. The G3X calculations showed these to be separated by 1.6 and 1.7 kJ mol^−1^, respectively. In addition, higher energy structures were, in most cases, predicted to be too stable, that is, the PES is predicted to be too flat. This is a significant problem for this work, where the higher energy structures are those of greatest interest and need to be described as accurately as possible. The RMS deviations were, however, significantly smaller than those calculated with either Amber or HF.

The MP2 calculations were again found to give by far the best agreement with the benchmarks. The RMS deviation was only 0.6 kJ mol^−1^ and the maximum deviation 1.2 kJ mol^−1^. MP2(full) was, therefore, chosen as the most reliable level of theory with which to calculate the 3D-PES.

### 3.2 The 3D-PES

**Figure 2:**
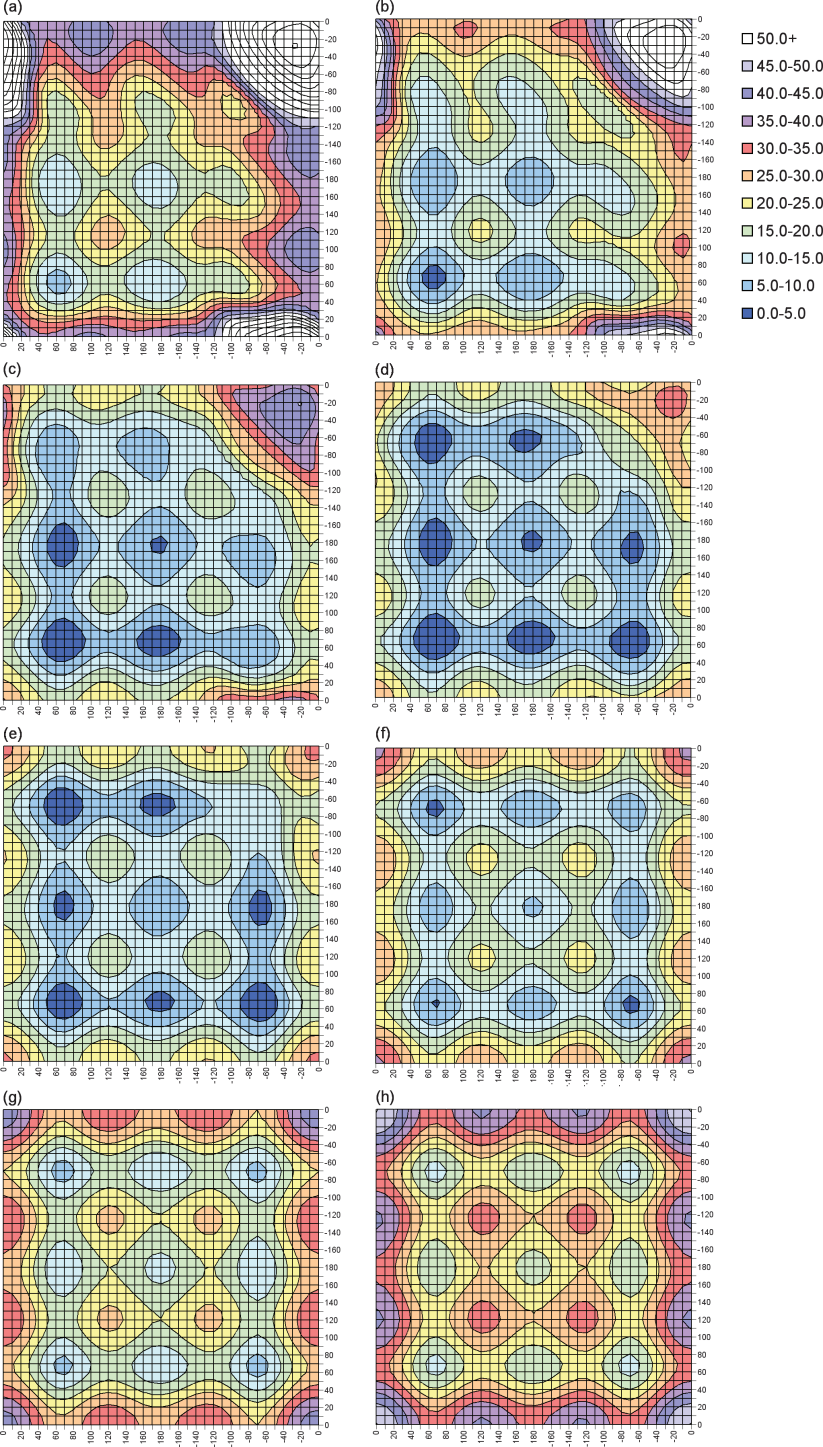
Contour plots of slices through the MP2(full)/6-31G(d) 3D-PES for DEDS. χ_3_ values are (a) 60°, (b) 70°, (c) 80°, (d) 90°, (e) 100°, (f) 110°, (g) 120° and (h) 130°. The horizontal and vertical axes show χ_2_ and χ_2_′. Due to the symmetry of the system, any specific labelling would be arbitrary. Energies, in kJ mol^−1^, are relative to the absolute minimum: χ_2_ = 70°, χ_3_ = 90° and χ_2_′ = 70°.

The PES is displayed in the form of contour plots for increasing values of χ_3_. Figure 2 shows slices through the surface for χ_3_ between 60 and 130°. Contour plots for χ_3_ values outside this range can be found in Appendix 1. Note that the contour plots for negative χ_3_ values are related by symmetry to those for χ_3_ > 0. In all contours the energies are shown relative to the energy of the absolute minimum of the 3D-PES, χ_2_ = 70°, χ_3_ = 90°, χ_2_′ = 70°. The colour scheme has been chosen so that blue regions are low in energy (<10 kJ mol^−1^). Disulfides with energies in this region are expected to be stable. Cyan indicates higher energy regions (energy between 10 and 15 kJ mol^−1^) and green represents very high energy regions (energy between 15 and 20 kJ mol^−1^). Disulfides in these regions are expected to be less stable and more likely to be involved in redox processes. All other colours correspond to extremely high energy regions (>20 kJ mol^−1^). Disulfides are not expected to be found with such high torsional strain.

At the lowest energy point on the surface, χ_3_ = 90°, the different minima reported by Görbitz [8] are seen as stable (dark blue) areas, with the saddle point regions between them also, in most cases, being of low energy (light blue). The area of the dark blue circles generally gives a good indication of the relative stability of the minima. Likewise the area of the green regions indicates the relative instability of the maxima in this slice. The plot appears largely as expected but, surprisingly, a minimum is not seen for χ_2_ ∼ − 60°, χ_3_ ∼ 90°, χ_2_′ ∼ − 60°, that is, for the G′GG′ conformation. Detailed checks of the relative energy of each gridpoint in the G′GG′ region confirm that the absence of this minimum is not an artefact of the positioning of the contours. The same situation is found for the contour with χ_3_ = 80°; while a very shallow minimum, with a depth of ∼1 kJ mol^−1^, is seen for χ_3_ = 100°. We note here that, whereas the χ_3_ values of the fully optimised benchmark calculations were approximately 90° for almost all conformations, for G′GG′ the value had increased to approximately 110° with all levels of theory. This unexpected instability of the G′GG′ region of the PES is due to steric clashes between the terminal methyl groups. In proteins, these correspond to the Hα atoms. As shown in figure 1, the Hα atoms of a G′GG′ (χ_2_ ∼ χ_2_′ ∼ − 60) disulfide are forced into particularly close proximity when χ_3_ = 90°. They, therefore, experience strong repulsive interactions and the system is destabilised. These steric interactions are reduced as χ_3_ moves away from 90°, thus allowing minima to appear for χ_3_ ≤ 70° and χ_3_ ≥ 110°. Interestingly, for χ_3_ = 70° the minimum is surprisingly deep (4.4 kJ mol^−1^), although it becomes shallower for smaller χ_3_ values.

The effects of steric interference are particularly important for lower values of χ_3_. When the χ_2_ and χ_2_′ dihedral angles are both small, the terminal methyl hydrogens come into very close contact as χ_3_ is reduced. This results in the high energy feature near the origin (actually at χ_2_ = χ_2_′ ∼ 20°) which grows rapidly as χ_3_ is reduced below 90°. The growth of this feature also has a significant adverse effect on the stabilities of the GGG′ and G′GT conformations for χ_3_ ≤ 70°. Although these minima are not seen on the contour plots for χ_3_ = 60 and 70°,they do exist. For both contours the minima are very shallow (∼2 kJ mol^−1^), but they become deeper again for χ_3_ < 50°. For χ_3_ < 40° the steric repulsion is so great that the entire contour plot lies in the extremely high energy region, above 20 kJ mol^−1^. Disulfides are not expected to occur in these regions.

As χ_3_ increases above 90°, the contour plot becomes more symmetrical due to the reduction of the steric interactions between the methyl groups. In particular, the GGG′ conformation drops in energy so that for χ_3_ = 100° it is equal in energy with GGG, and for χ_3_ = 110 and 120° it is actually the most stable conformation on the PES. When χ_3_ is increased to 120° there is no longer steric strain in the G′GG′ region so that the G′GG′ conformation is now of equal stability to GGG. The PES continues to look effectively symmetrical for all higher values of χ_3_. For χ_3_ values of 150° and above, the entire surface is more than 20 kJ mol^−1^ above the minimum. Again, disulfides with these large χ_3_ values are not expected to exist.

For comparison, contour plots of the Amber force field 3D-PES are shown in Appendix 2. Note that the absolute minimum on the Amber PES is at χ_2_ = 180°, χ_3_ = 80°, χ_2_′ = 180°, rather than at χ_2_ = 70°, χ_3_ = 90°, χ_2_′ = 70° as seen with MP2(full). However, all energies are shown relative to the MP2(full) absolute minimum to give the clearest comparison between the two methods. Areas shown in very dark blue lie below the MP2(full) minimum. We note, however, that the difference between χ_2_ = 180°, χ_3_ = 80°, χ_2_′ = 180° and χ_2_ = 70°, χ_3_ = 90°, χ_2_′ = 70° on the Amber PES is only 1.5 kJ mol^−1^.

All the significant features seen in the MP2(full) 3D-PES are also found in the Amber force field surface, albeit shifted to slightly lower χ_3_ angles. The Amber PES does, however, seem to be rather flatter than the MP2(full) version, with the energy not rising as quickly as χ_3_ moves away from 90° (even when the difference in reference energy is taken into account). The most significant discrepancy, is in the prediction of the relative stabilities of the local minima. Comparison of figures 2 and A2 (Appendix 2) clearly shows, amongst other problems, an inversion of the order of the GGG, GGT and TGT conformations for most values of χ_3_. This issue has already been discussed in detail in Section 3.1.

### 3.3 Comparison of PES with observed disulfide conformations

**Figure 3:**
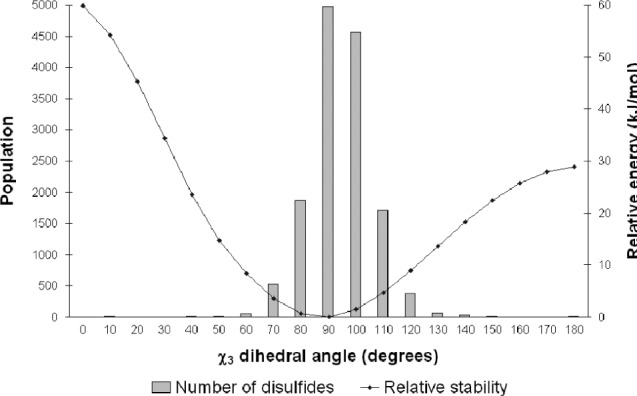
The variation of disulfide population with torsion around the χ_3_ dihedral angle, as obtained from the database of high resolution X-ray structures. The change in energy associated with this torsion for the GGG conformation (χ_2_ = 60°, χ_3_ varied, χ_2_′ = 60°) is also shown.

An important test of the usefulness of our PES is to check that the disulfide conformations that are predicted to be the most stable actually correspond to those most commonly seen in proteins. Figure 3 shows the distribution of χ_3_ values obtained from our database of high resolution X-ray structures. Superimposed on this distribution is the (one-dimensional) PES for torsion of χ_3_ in the most populous GGG conformation. This gives a rough guide as to how the shape of the 3D-PES changes with χ_3_. We note that the 1D-PESs for most other conformations have the same general shape, although the minima may be at slightly different dihedral angles. It can be seen that the most stable point on the PES, at about 90°, does indeed correspond to the highest population of disulfides. The population falls rapidly as χ_3_ increases or decreases and the energy rises. The slight shift of the histogram towards higher χ_3_ values is probably associated with the minima for some conformations being as high as 110° (G′GG′).

**Figure 4:**
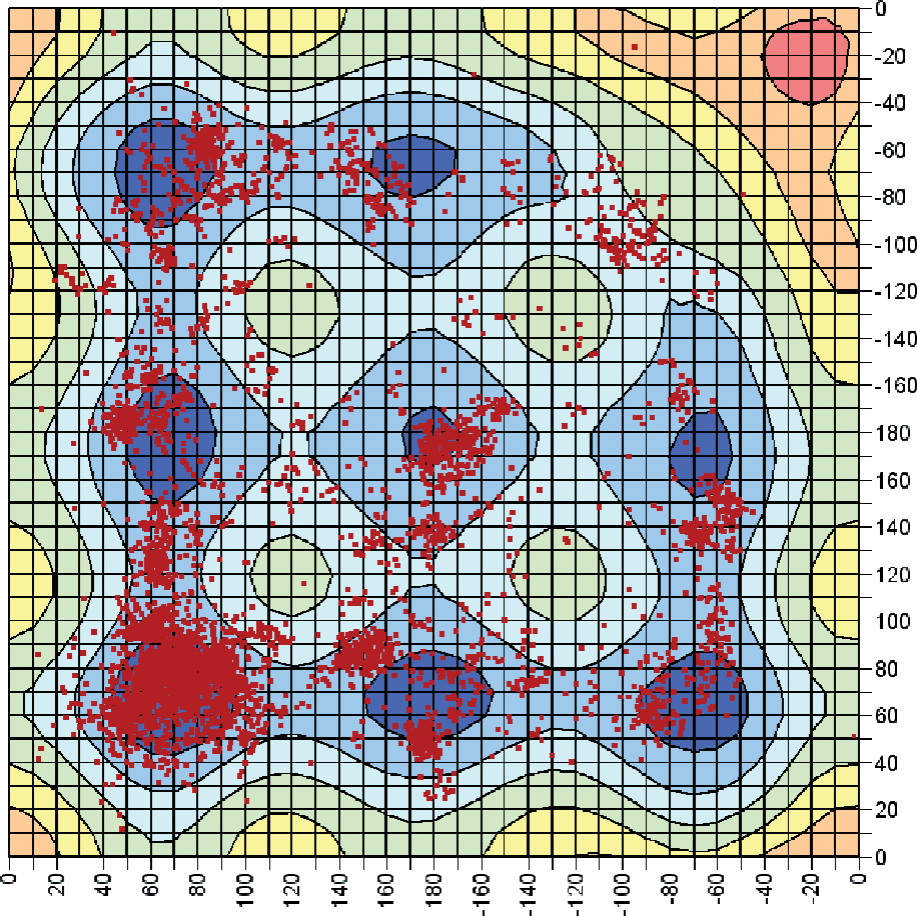
Scatter plot of experimental χ_2_ and χ_2_′ values for disulfides from the database of high resolution X-ray structures with χ_3_ between 85 and 95° superimposed on the 3D-PES slice for χ_3_ = 90°.

It is also interesting to compare how the disulfides in the PDB are distributed amongst the possible conformations. Figure 4 shows a scatter plot of the conformations adopted by disulfides with χ_3_ between 85 and 95°. This is superimposed on the PES slice for χ_3_ = 90°. Clearly the majority of the disulfides are located in either the low energy (blue) or high energy (cyan) regions, with very few examples in the very high energy zones (green, yellow, etc.). By far the majority of the disulfides adopt the lowest energy GGG conformation (bottom LH corner). However, concentrated patches are seen for each of the different minima. We note that for χ_3_ = 90° the saddle points are also in the low energy region of the PES and significant populations of disulfides are found with the corresponding sets of dihedral angles.

What is most interesting is that an appreciable number of disulfides is also seen in the high energy G′GG′ region (top RH corner). Further analysis of the structures which adopt this conformation has revealed that, in almost all cases, the disulfide is fixed in this conformation by the protein secondary structure. In particular, most of these disulfides are found to bridge two neighbouring strands in an antiparallel β-sheet. This secondary structure motif is known as a cross-strand disulfide [4,18,20]. In addition to the strain due to the unfavourable conformation, the associated β-sheets are also significantly distorted by the presence of these disulfides. They are, therefore, highly strained and present very promising candidates for involvement in redox processes. Further analysis of this interesting class of disulfides has been reported elsewhere [5].

### 3.4 Use of the 3D-PES to predict disulfide stability

Finally, the 3D-PES was used to predict the strain in each of the disulfide bonds found in our database of high resolution structures from the Protein Data Bank. This was done using a simple three-dimensional linear interpolation on the calculated PES. The effects of strain in the χ_1_ and χ_1_′ dihedral angles were not taken into account in this investigation.

Using our MP2(full) PES, the mean strain energy of the disulfides in our database was found to be 7.1 kJ mol^−1^, with a standard deviation of 4.8 kJ mol^−1^. Seventy-nine percent of disulfides were found to have relatively low energy (<10 kJ mol^−1^ above the minimum), with a further 18% being in the high energy region (between 10 and 15 kJ mol^−1^). Only 3% had a relative energy higher than 15 kJ mol^−1^.

**Figure 5:**
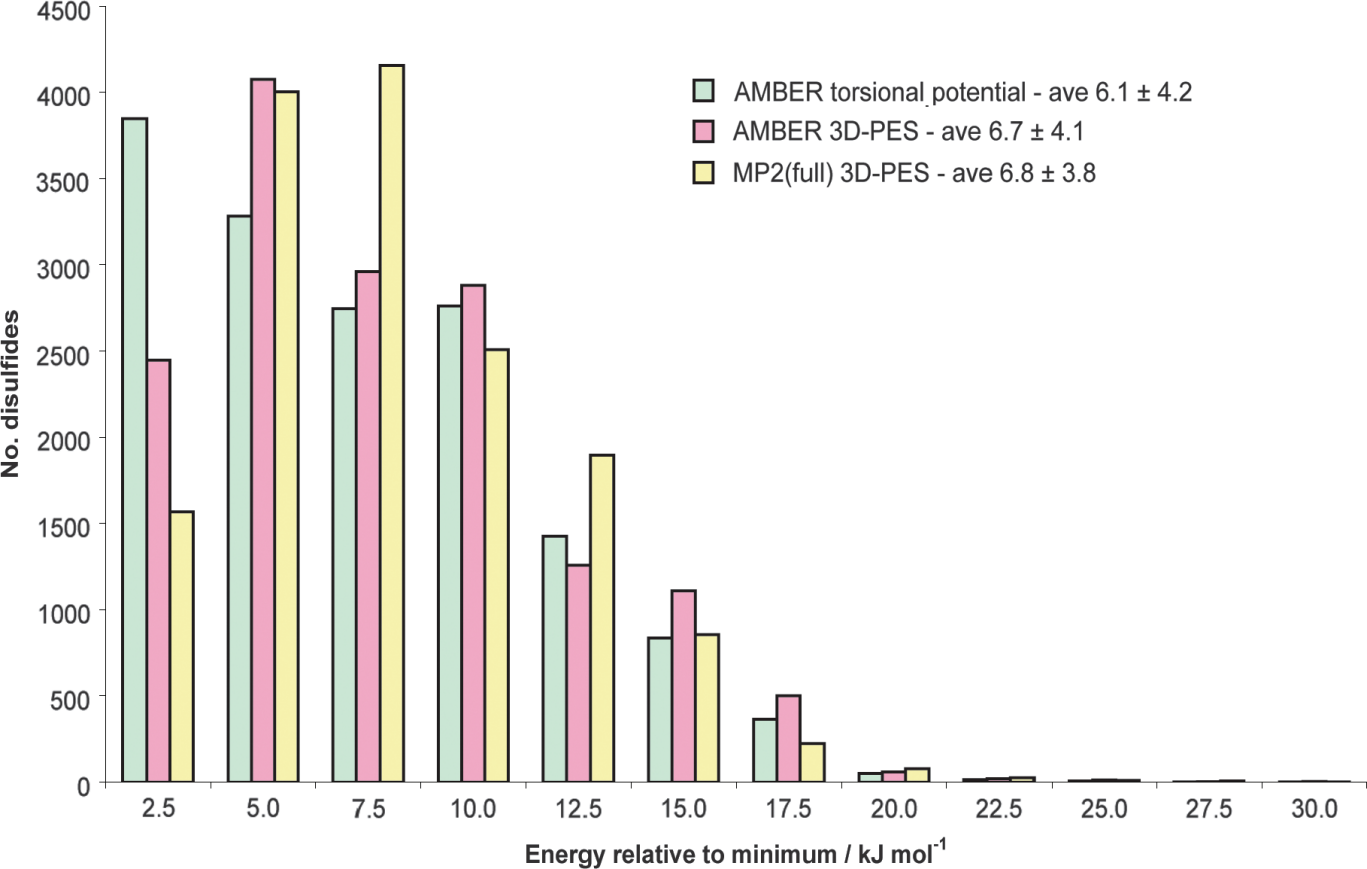
Comparison of relative energies for disulfides in high resolution structures of the PDB as predicted by the (a) Amber torsional potential, (b) full Amber potential including non-bonded terms, and (c) quantum chemical calculations using the MP2(full) level of theory. Note particularly the populations peak in different energy bins. To ensure a fair comparison, only disulfides with χ_3_ between 60 and 130° were included (see Methods).

A histogram showing the energy distribution of the disulfides in the PDB can be found in figure 5. Energy distributions calculated using the Amber torsional potential and the Amber 3D-PES have been included in addition to the MP2(full) results. The average disulfide energies are also shown along with the standard deviations. As discussed in Methods, only disulfides with χ_3_ between 60 and 130° are included, resulting in a slightly different MP2(full) average compared to that for the complete dataset stated above. Using energies calculated with the Amber torsional potential, the histogram is seen to be strongly biased towards lower energies; this is a natural result of the prediction by this potential that all the configurations on the PES are equally stable and thus all have a relative energy of zero. Using the energies from the Amber 3D-PES, the histogram is much more consistent with that from the MP2(full) surface, but is still biased towards lower energies. This is clearly seen by examining the modal energies for each method. The histogram peaks at 2.5 kJ mol^−1^ if only the Amber torsional energy is considered; at 5.0 kJ mol^−1^ when the entire Amber potential is considered; and at 7.5 kJ mol^−1^ using the more exact MP2(full) calculations.

Further analysis of the relative energies associated with each of the different disulfide conformations as well as with various secondary structure elements will be reported elsewhere [18].

## 4. Conclusions

We have successfully constructed a MP2(full)/6-31G(d) PES for the torsion of DEDS around its three important dihedral angles. This surface was found to be qualitatively different from that which was predicted using either the Amber torsional energy function or the full Amber force field. In particular, the relative stabilities of the minima on the MP2(full) surface were found to be in good agreement with the G3X benchmark calculations, whereas the Amber force field gave not only large deviations in the relative energies but also a different order for the stabilities of the conformations. This order is likely to be important in elucidating the mechanisms of reactions that involve a cascade of disulfides. One such example occurs in *Staphylococcus aureus* Arsenate reductase, in which stepwise formation of the Cys 10–Cys 82 and Cys 82–Cys 89 disulfides form part of the reaction cycle to detoxify arsenic [21]. The Cys 10–Cys 82 disulfide is a short-lived high energy intermediate trapped in the crystal structure of the Cys89Leu mutant (PDB 1lk0). Upon formation, the disulfide likely adopts the SG′T conformation with a relative energy of 14.9 kJ mol^−1^ (Chain A). Subsequent movements of a flexible region of the backbone (residues 82–97) twist the disulfide into an S′GG conformation with a relative energy of 24.0 kJ mol^−1^, rendering it susceptible to attack by the nearby Cys 89 thiolate (Chain B). The subsequently formed Cys 82–Cys 89 disulfide adopts the G′G′G′ conformation with a lower energy of 12.2 kJ mol^−1^, and is sufficiently stable that it must be reduced by thioredoxin in order to regenerate Arsenate reductase for the next reaction cycle (PDB 1lju).

The relative configurational stabilities of the MP2(full) PES were also found to be more consistent with experimental data for the populations of disulfides, which adopt the associated conformations.

Unexpectedly, the 3D-PES did not show a minimum associated with the G′GG′ conformation for χ_3_ values of 80 and 90°. This is a result of strong steric interactions with this particular set of dihedral angles (χ_2_ ≈ χ_2_′ ≈ − 60°, χ_3_ ≈ 90°). In this conformation the Hα atoms are aligned directly towards each other, thus experiencing strong repulsive forces that destabilise the system. Also surprising was that a significant population of disulfides were found to adopt this high energy conformation. Further analysis revealed that in most cases this conformation arose from (and was required for) an unusual secondary structure motif, the cross-strand disulfide.

The 3D-PES was subsequently used to predict the relative stabilities of all the high resolution disulfide bonds reported in the Protein Data Bank. As expected, the vast majority of the disulfides were found to have a low strain energy and are, therefore, likely to be involved solely in structural stabilisation. Approximately 20% of the cystines were of high or very high relative energy and thus have the potential to be involved in redox processes. Further investigation of these disulfides is ongoing.

## Appendix 1

Contour plots of slices through the MP2(full)/6-31G(d) 3D-PES for DEDS. χ_3_ values are: (a) 0°, (b) 10°, (c) 20°, (d) 30°, (e) 40°, (f) 50°, (g) 140°, (h) 150°, (i) 160°, (j) 170° and (k) 180°. The horizontal and vertical axes show χ_2_ and χ_2_′. Due to the symmetry of the system, any specific labelling would be arbitrary. Energies, in kJ mol^−1^, are relative to the absolute minimum: χ_2_ = 70°, χ_3_ = 90°, χ_2_′ = 70° (Figure A1a–k)

## Appendix 2

Contour plots of slices through the Amber force field 3D-PES for DEDS. χ_3_ values are (a) 60°, (b) 70°, (c) 80°, (d) 90°, (e) 100°, (f) 110°, (g) 120° and (h) 130°. The horizontal and vertical axes show χ_2_ and χ_2_′. Due to the symmetry of the system, any specific labelling would be arbitrary. Energies, in kJ mol^−1^, are relative to χ_2_ = 70°, χ_3_ = 90°, χ_2_′ = 70°, the minimum on the MP2(full) PES (Figure A2a–h)
